# Association of baseline and dynamic arterial stiffness status with dyslipidemia: a cohort study

**DOI:** 10.3389/fendo.2023.1243673

**Published:** 2023-11-23

**Authors:** Hai Wang, Zhanhao Mo, He Sui, Yitian Qi, Peiwen Xu, Jia Zheng, Te Zhang, Xin Qi, Cancan Cui

**Affiliations:** China-Japan Union Hospital of Jilin University, Jilin University, Jilin, China

**Keywords:** arterial stiffness, brachial-ankle pulse wave velocity, dyslipidemia, lipid profile, cohort study

## Abstract

**Background and aims:**

Dyslipidemia is known to contribute to arterial stiffness, while the inverse association remains unknown. This study aimed to explore the association of baseline arterial stiffness and its changes, as determined by brachial-ankle pulse wave velocity (baPWV), with dyslipidemia onset in the general population.

**Methods:**

This study enrolled participants from Beijing Health Management Cohort using measurements of the first visit from 2012 to 2013 as baseline, and followed until the dyslipidemia onset or the end of 2019. Unadjusted and adjusted Cox proportional regression models were used to evaluate the associations of baseline baPWV and baPWV transition (persistent low, onset, remitted and persistent high) with incident dyslipidemia.

**Results:**

Of 4362 individuals (mean age: 55.5 years), 1490 (34.2%) developed dyslipidemia during a median follow-up of 5.9 years. After adjusting for potential confounders, participants with elevated arterial stiffness at baseline had an increased risk of dyslipidemia (HR, 1.194; 95% CI, 1.050-1.358). Compared with persistent low baPWV, new-onset and persistent high baPWV were associated with a 51.2% and 37.1% excess risk of dyslipidemia.

**Conclusion:**

The findings indicated that arterial stiffness is an early risk factor of dyslipidemia, suggesting a bidirectional association between arterial stiffness and lipid metabolism.

## 1 Introduction

Dyslipidemia refers to the metabolic disorder of lipoprotein in human body, which mainly includes the increase of total cholesterol, low-density lipoprotein (LDL) cholesterol, triglycerides and the decrease of high-density lipoprotein (HDL) cholesterol. Dyslipidemia is one of the important factors leading to atherosclerosis (1) and an independent risk factor for coronary heart disease (2) and ischemic stroke (3). The global burden of dyslipidemia has increased over the past 30 years, and LDL cholesterol has ranked the 8th leading risk factor of mortality in 2019 (4). The Global Burden of Disease Report (1990-2019) showed that the total number of disability adjusted life years (DALYs) due to LDL cholesterol reached 98.6 million (95% UI: 80.3 to 119.0 million), and there were 4.4 million (95% UI: 3.3 to 5.7 million) deaths in 2019 (5). According to the 2007-2014 National Health and Nutrition Examination Survey (NHANES) data, the overall prevalence of hypertriglyceridemia was 25.9% (6). The age-standardized prevalence of dyslipidemia among people aged above 35 years in northern China from 2015 to 2017 was 31.2% (7). Thus, the early detection and management of dyslipidemia is needed to improve the life quality, which requires novel predictors for risk stratification beyond the identified risk factors, including age (8), obesity (9), lifestyle (10), family history of disease (11) and genetics factors (12).

Arterial stiffness reflects the abnormality of arterial structure and function. Arterial stiffness can cause damage to multiple organs, including heart, brain, and kidney, resulting in coronary heart disease (13), hypertension (14, 15), acute ischemic stroke (16), chronic kidney disease (17), thus is strongly associated with an increased risk of all-cause mortality (18). Several noninvasive methods of detecting arterial stiffness have been developed, such as the pulse wave velocity (PWV) (19). Diabetes (20) and dyslipidemia (21) are thought to alter the arterial stiffness level. On the other hand, two recent studies (22, 23) showed that arterial stiffness is also reversely associated with the incidence of diabetes, and even precedes the development of diabetes. Nakano et al., concluded that there exists bidirectional association between arterial stiffness and hypertension (24). Previous studies have confirmed that dyslipidemia (e.g., low HDL cholesterolemia and high LDL cholesterolemia) is associated with an increased risk of arterial stiffness (25, 26). However, the temporal relationship from arterial stiffness to dyslipidemia remains unknown. Given the effect of baseline arterial stiffness level on the progression of triglyceride in adolescence and young adulthood in a recent study (27), we hypothesized arterial stiffness is also a risk factor of dyslipidemia development in the general population.

Therefore, the objective of this present study is to explore the associations of baseline arterial stiffness and the dynamic transition of arterial stiffness status with the incidence of dyslipidemia in adults. This is the first to our best knowledge that investigates the reverse relationship of arterial stiffness with incident dyslipidemia using a cohort design, which would contribute to the systematic understanding of the bidirectional association between arterial stiffness and lipids metabolism.

## 2 Methods and materials

### 2.1 Study cohort and settings

We recruited participants from Xiaotangshan Health Examination Center, which is based on the health examination population and aims to collect and investigate the risk factors and biomarkers of cardiometabolic diseases. Details of the cohort design have been described in previous study (28, 29). In brief, the participants undertaking physical health examinations were included in this dynamic cohort and repeated annual follow-up. All participants provided written informed consent before taking part in this study. The study data were anonymously analyzed and the study was approved by the Ethics Committee of Beijing Xiaotangshan Hospital.

**Figure 1:**
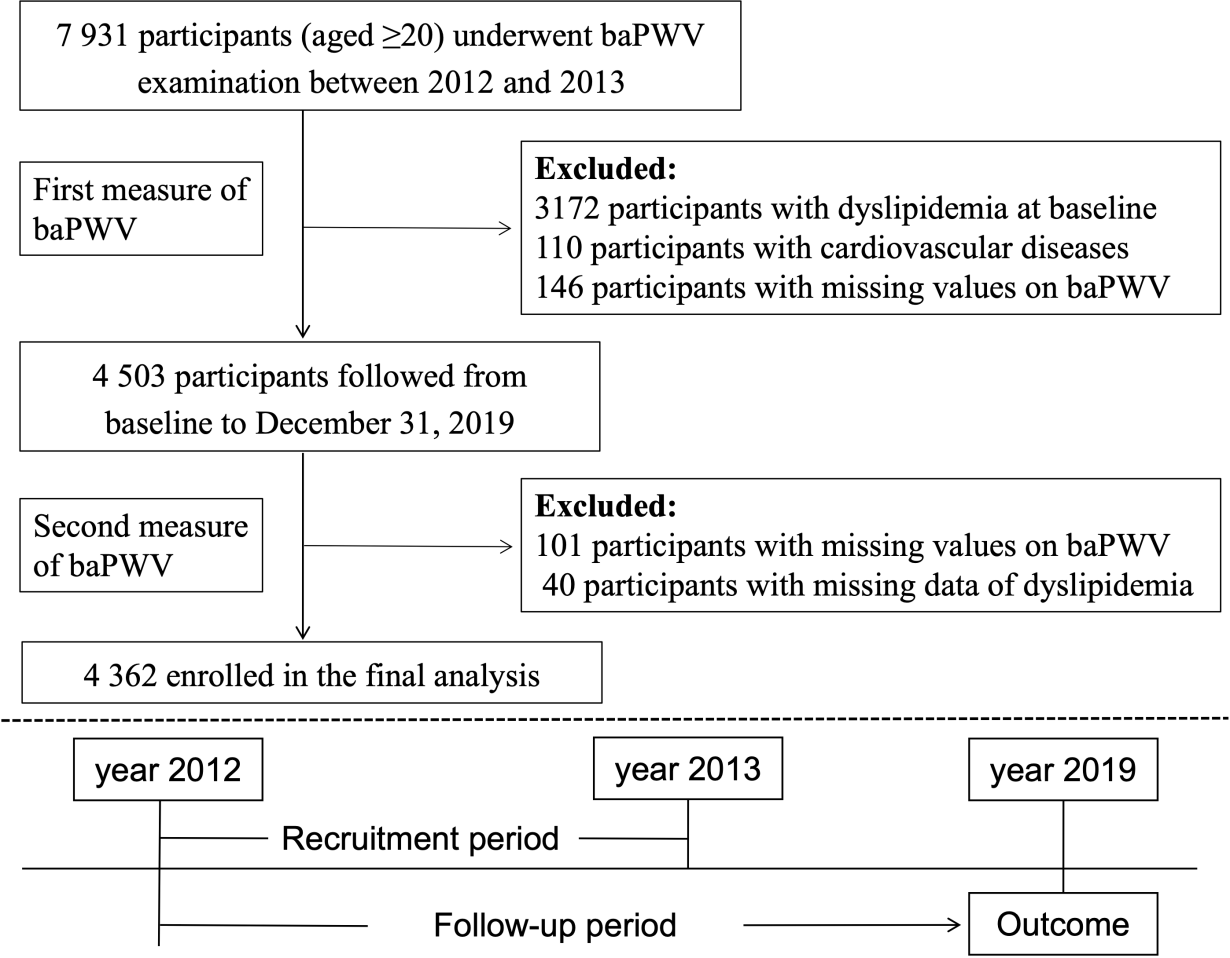
Flowchart of this current study.

This current study used the baPWV data of the first visit from 2012 to 2013 as baseline and followed up until the development of dyslipidemia or the end of study period (December 31 of 2019), which came first. We excluded participants who already had dyslipidemia (n=3172), self-reported cardiovascular diseases (n=110) which could alter the arterial stiffness level, or missing baPWV data (n=146) at baseline. Participants missing baPWV data or dyslipidemia information (n = 141) at follow-up were further excluded. Thus, the final analysis cohort consisted of a subset of 4 362 participants (**Figure 1**).

### 2.2 Data collection and definition

Face-to-face interviews were annually conducted using standardized questionnaires to collect the demographic characteristics (e.g. age and sex), lifestyles (e.g. smoking status, alcohol consumption and physical activity), diagnosis history of diseases and medication use information. In detail, smoking status was divided into “never” “former” and “current”. Drinking status was divided into “current drinking” and “no current drinking”. Physical activity was defined as “≥80 minutes of moderate or vigorous physical activity per week”. Disease history included the self-reported cardiovascular diseases, hypertension, diabetes, and dyslipidemia.

Physical examination parameters include height, weight and blood pressure. Body mass index (BMI) was calculated as the weight (kg)/height (m)^2^. Obesity was defined as BMI ≥28.0 kg/m^2^. Blood pressure was measured in the seated position using a mercury sphygmomanometer, and the average value of three readings was recorded as systolic blood pressure (SBP) and diastolic blood pressure (DBP). Mean arterial blood pressure (MAP) was calculated as (SBP+2*DBP)/3 (30).

Blood samples from the participants were analyzed using the Olympus Automatic Biochemical Analyser (Hitachi 747; Tokyo, Japan). Biochemical parameters included fasting glucose, triglycerides, total cholesterol, high-density lipoprotein (HDL) cholesterol, low-density lipoprotein (LDL) cholesterol. NonHDL cholesterol was defined as total cholesterol minus HDL cholesterol (31). Remnant cholesterol was calculated by Martin-Hopkins equation (32).

### 2.3 Arterial stiffness and dyslipidemia

BaPWV was measured using the Omron Colin BP-203RPE III device (Omron Health Care, Kyoto, Japan). BaPWV has been widely used in clinical practice and large population studies, and it reflects the arterial system with high compliance and reproducibility. The detailed process has been described in a previous study (33). BaPWV was both measured at baseline and follow-up. The maximum value of baPWV on the left and right sides was selected as the final value of arterial stiffness level. After resting in supine position for 5 minutes, 4 cuffs were wrapped around the bilateral brachia and ankles and then connected to a plethysmographic sensor and oscillometric pressure sensor. The elevated arterial stiffness was defined as baPWV ≥1400cm/s.

According to the Guidelines of Prevention and Treatment of Dyslipidemia for Chinese Adults (34), dyslipidemia was defined as triglycerides ≥ 2.3 mmol/L or total cholesterol ≥ 6.2 mmol/L or LDL cholesterol ≥ 4.1 mmol/L or HDL cholesterol < 1.0 mmol/L or use of lipid-lowering medication.

### 2.4 Statistical analysis

Continuous variables with normal and skewed distributions were described as the mean with standard deviation (SD) and the median with interquartile range (IQR), which were compared using Student’s t-test and Mann-Whitney U test, as appropriate. Categorical variables were described as numbers and proportions, compared using Chi-square test.

Individuals were grouped according to clinical cutoff point (1400 cm/s) and quartile values of baseline baPWV level. Based on the two measurements of baPWV at baseline and follow-up, participants were divided into four groups: persistent low baPWV, transit to high baPWV from low level (onset), remit to low baPWV from high level (remitted) and persistent high baPWV. As a cohort design with annual follow-up data, cox proportional hazards regression model was used to estimate the associations of baseline baPWV and baPWV transition with the incidence of dyslipidemia after satisfying the PH assumption. Hazard ratios (HR) with 95% confidence interval (CI) were then calculated. We adjusted potential confounders in the Cox regression models. Model 1 was adjusted for age and sex; model 2 was further adjusted for obesity, fasting glucose, diabetes, hypertension, physical activity, smoking status and drinking status. We carried out restricted cubic spline analysis to explore the dose-response relationship between baseline baPWV and the risk of dyslipidemia development using three knots at the 10th, 50th, and 90th percentiles, which is a widely used visualization method to depict the nonlinear relationship between exposure and outcome. In addition, we analyzed and visualized the effect of baPWV change on lipid parameter progression during follow-up using partial correlation analysis with age and sex adjusted. The multi-variable linear regression model then was used to evaluate the association between baPWV change (per 100 cm/s) and the progression of lipid parameters.

All statistical analyses were performed using R software (version 4.1.0), and a two-sided P value < 0.05 was considered statistically significant.

## 3 Results

### 3.1 Baseline characteristics

The final analysis included 4 362 individuals, and the mean age of the population was 55.53 (13.94) years. During the follow-up period, 1 490 participants developed dyslipidemia, including 1 119 males (75.1%) and 371 females (24.9%). **Table 1** shows the baseline characteristics of individuals of incident dyslipidemia or not. Compared with participants free of dyslipidemia, those developing dyslipidemia had higher age, BMI, MAP, fasting glucose, LDL cholesterol and baPWV. We also analyzed the baseline characteristics stratified by baPWV level (**Supplementary File 1**: **Supplementary Table S1**). The cumulative incidence rate of dyslipidemia in participants with baPWV ≥ 1400 cm/s at baseline was 39.0% (850/2180), which was higher than those with baPWV < 1400 cm/s (29.3%; 640/2182).

**Table 1:** Characteristics of participants.

Characteristics	Overall	No Dyslipidemia	Incident Dyslipidemia
Participants, No.	4362	2872	1490
Age, years	55.53 (13.94)	54.40 (14.38)	57.72 (12.79)
-39 years	437 (10.0)	350 (12.2)	87 (5.8)
40-59 years	2480 (56.9)	1671 (58.2)	809 (54.3)
60- years	1445 (33.1)	851 (29.6)	594 (39.9)
Male, n (%)	2993 (68.6)	1874 (65.3)	1119 (75.1)
BMI, kg/m^2 a^	25.02 (3.09)	24.79 (3.08)	25.48 (3.04)
Obesity, n (%)	681 (15.9)	411 (14.5)	270 (18.5)
Physical activity (n, %) ^b^	2082 (47.7)	1369 (47.7)	713 (47.9)
Smoking status (n, %)			
Never	3298 (75.6)	2184 (76.0)	1114 (74.8)
Former	515 (11.8)	324 (11.3)	191 (12.8)
Current	549 (12.6)	364 (12.7)	185 (12.4)
Current drinking (n, %)	2817 (64.6)	1868 (65.0)	949 (63.7)
Hypertension, n (%)	352 (8.1)	212 (7.4)	140 (9.4)
Diabetes, n (%)	119 (2.7)	71 (2.5)	48 (3.2)
MAP, mmHg ^c^	106.21 (13.41)	105.22 (13.28)	108.10 (13.46)
Fasting glucose, mmol/L	5.47 (1.08)	5.42 (1.09)	5.57 (1.05)
Triglycerides, mg/dl	101.00[75.31,134.67]	93.03[70.88,122.27]	121.38[89.49,154.16]
Total cholesterol, mg/dl	180.93[161.99,199.10]	179.00[160.83,196.01]	187.11[164.69,206.44]
LDL-C, mg/dl	116.75[97.42,133.38]	113.66[96.26,129.51]	123.33[101.87,141.11]
HDL-C, mg/dl	49.87[43.69,57.60]	51.03[45.23,58.76]	46.39[41.75,54.51]
Non HDL-C, mg/dl ^d^	129.12[109.41,146.91]	124.87[106.70,142.27]	137.63[115.21,155.03]
Remnant cholesterol, mg/dl ^e^	19.98[16.51,24.57]	18.96[15.87,22.69]	22.66[18.69,26.73]
baPWV, cm/s	1399[1266,1620]	1376[1244,1590]	1440[1307,1663]
baPWV ≥1400 cm/s, n (%)	2180 (50.0)	1330 (46.3)	850 (57.0)

Continuous data are presented as mean (SD) or median [IQR], as appropriate.

BMI, body mass index; MAP, mean arterial pressure; baPWV, brachial-ankle pulse wave velocity; LDL-C, low-density lipoprotein cholesterol; HDL-C, high-density lipoprotein cholesterol.

^a^BMI is calculated as weight in kilograms divided by height in meters squared; ^b^Physical activity refers to having moderate or intense exercise ≥80 minutes a weak; ^c^MAP is calculated as (systolic blood pressure+2×diastolic blood pressure)/3; ^d^Non HDL-C refers to total cholesterol minus HDL-C; ^e^Remnant cholesterol is calculated by Martin-Hopkins equation.

### 3.2 Association between arterial stiffness and dyslipidemia

**Figure 2:**
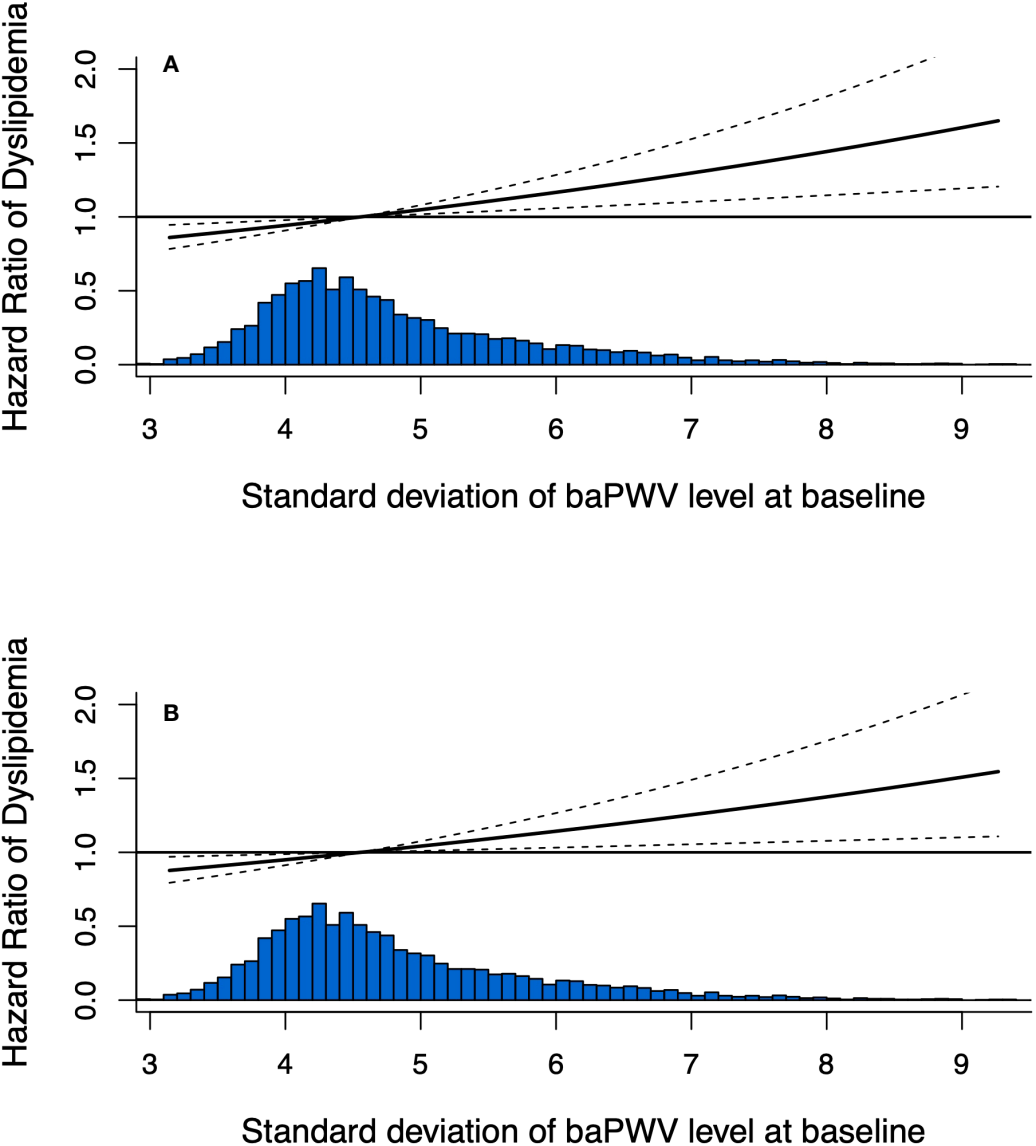
Dose-response relationship between baseline baPWV and the development of dyslipidemia using restricted cubic spline method. Restricted cubic spline regression model was conducted using 3 knots at the 10th, 50th, and 90th percentiles; **(A)** adjusted for age and sex; **(B)** adjusted for age, sex, obesity, fasting glucose, diabetes, hypertension, physical activity, smoking status and drinking status.

In the fully adjusted model (model 2), the HR for developing dyslipidemia was 1.097 (95% CI, 1.022-1.177) for per-SD increase of baPWV. Compared with baPWV <1400cm/s, those of baPWV ≥1400cm/s were significantly associated with an increased risk of dyslipidemia onset (HR, 1.194; 95% CI, 1.050-1.358). The HR values of dyslipidemia were 1.000 (reference), 1.497 (95% CI, 1.269-1.766), 1.519 (95% CI, 1.279-1.803) and 1.550 (95% CI, 1.164–1.899) for the quartile groups of baPWV (*P* for trend <.001) (**Table 2**). In addition, the observed associations were consistent among those <60 and ≥60 years as shown in **Supplementary File 1** (**Supplementary Table S2**). Arterial stiffness is consistently associated with dyslipidemia risk when alternatively defined by using lipid-lowering therapy (**Supplementary File 1**: **Supplementary Table S3**). Furthermore, the interactive effect between inclusion period (2012-2014 or 2015-2017) and arterial stiffness group on the onset of dyslipidemia was insignificant under a dynamic cohort design. Moreover, the restricted cubic spline curves showed a significant dose-response relationship between baseline baPWV and dyslipidemia onset (**Figure 2**).

**Table 2:** Association between baseline baPWV and the development of dyslipidemia.

	Dyslipidemia No./Total No.	Hazard Ratio (95% CI)			
		model 1	*P* value	model 2	*P* value
baPWV, per-SD increase		1.112(1.04-1.189)	.002	1.097(1.022-1.177)	.01
Two groups					
baPWV <1400 cm/s	640/2182	ref		ref	
baPWV ≥1400 cm/s	850/2180	1.251(1.107-1.415)	<.001	1.194(1.050-1.358)	.007
Quartile groups					
-1266 cm/s	260/1096	ref		ref	
1267-1399 cm/s	380/1086	1.503(1.280-1.763)	<.001	1.497(1.269-1.766)	<.001
1400-1620 cm/s	422/1090	1.589(1.347-1.873)	<.001	1.519(1.279-1.803)	<.001
1621- cm/s	428/1090	1.623(1.337-1.971)	<.001	1.550(1.264-1.899)	<.001

CI, confidence interval; baPWV, brachial-ankle pulse wave velocity.

model 1: adjusted for age and sex; model 2: adjusted for age, sex, obesity, fasting glucose, diabetes, hypertension, smoking, drinking and physical activity.

### 3.3 Arterial stiffness change and dyslipidemia

The characteristics according to the baPWV status at baseline and follow-up were shown in **Supplementary File 1** (**Supplementary Table S4**). The cumulative incidence of dyslipidemia among persistent low baPWV, new-onset high baPWV, remitted low baPWV and persistent high baPWV were 25.8%, 39.0%, 35.2% and 39.4%, respectively. Compared with persistent low baPWV, new-onset and persistent high baPWV were associated with a 51.2% and 37.1% excess risk of dyslipidemia, while remitted low baPWV was not (**Table 3**).

**Table 3:** Association between dynamic transition of arterial stiffness status and dyslipidemia onset.

	Dyslipidemia No./Total No.	Hazard Ratio (95% CI)			
		model 1	*P* value	model 2	*P* value
baPWV<1400 cm/s at baseline → baPWV<1400 cm/s at follow-up	414/1602	ref		ref	
baPWV<1400 cm/s at baseline → baPWV≥1400 cm/s at follow-up	226/580	1.661(1.351-2.040)	<.001	1.512(1.220-1.871)	<.001
baPWV≥1400 cm/s at baseline → baPWV<1400 cm/s at follow-up	76/216	1.423(1.047-1.922)	.02	1.356(0.985-1.855)	.06
baPWV≥1400 cm/s at baseline → baPWV≥1400 cm/s at follow-up	774/1964	1.486(1.239-1.781)	<.001	1.371(1.134-1.658)	.001

CI, confidence interval; baPWV, brachial-ankle pulse wave velocity.

model 1: adjusted for age and sex; model 2: adjusted for age, sex, obesity, fasting glucose, self-reported diabetes, self-reported hypertension, smoking, drinking, and physical activity.

### 3.4 Lipid parameters progression

**Figure 3:**
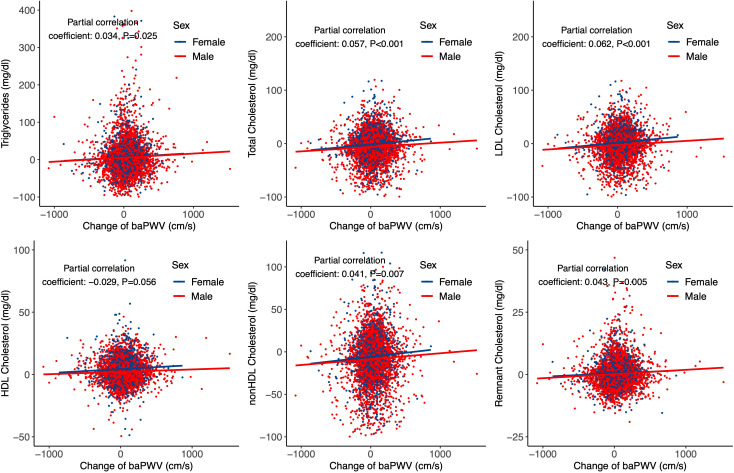
Partial correlation and regression lines between baPWV change and lipid parameters progression. Partial correlation coefficients were adjusted for age and sex.

In addition, we used partial correlation analysis and linear regression model to analyze the effect of baPWV changes on lipid parameter progression during follow-up. Partial correlation analysis showed that baPWV changes were associated with all lipid parameters except HDL cholesterol (**Figure 3**). Consistent results were observed in the linear regression model after adjusting for age, sex, obesity, fasting glucose, self-reported diabetes, self-reported hypertension, smoking, drinking, and physical activity (**Supplementary File 1**: **Supplementary Table S5**).

## 4 Discussion

In this population-based cohort study, we found that initial high arterial stiffness level determined by baPWV is independently associated with the incidence risk of dyslipidemia. We further observed that new-onset and persistent high baPWV are significantly associated with dyslipidemia development during follow-up, but not the remitted baPWV. The effect of high baPWV level on the onset of dyslipidemia was independent after adjusting for the most recognized risk factors of dyslipidemia.

Previous studies have reported the risk factors of arterial stiffness. A cohort study (35) showed that hyperglycemia, Hb1Ac, hypertension, and dyslipidemia are all determinants of arterial stiffness. Genetic study (36) also proved that hereditary elevated fasting plasma glucose level is associated with arterial stiffness. The triglyceride glucose (TyG) index, an indicator of insulin resistance, is also associated with arterial stiffness. Cohort studies (37–39) reported that participants with a higher TyG index are more likely to have a higher risk of arterial stiffness. In addition, various lipid markers are also closely related with arterial stiffness. Several studies (40–42) have shown that the total cholesterol, non-HDL-C and triglycerides are positively correlated with baPWV, while HDL-C was negatively associated with arterial stiffness (43, 44). Dyslipidemia is a potential risk factor for arterial stiffness even among those with hypertension (45). A secondary analysis based on a cross-sectional study reported that lipid variates are associated with arterial stiffness, and triglycerides levels are positively related to arterial stiffness, even independent of cardiovascular risks and liver function (46). Beyond, increased remnant cholesterol could be a significant predictor of arterial stiffness (47). In addition, a meta-analysis demonstrates that statin therapy has a beneficial effect on aortic arterial stiffness (48, 49).

A Swedish study (50) showed that cardiorespiratory fitness is negatively associated with arterial stiffness in young people. An observational (51) and mendelian randomized study (52) has reported a positive and causal relationship between obesity and arterial stiffness. Endothelial and vascular smooth muscle cell stiffness, extracellular matrix remodeling, perivascular adipose tissue inflammation and immune cell dysfunction are developmental factors of arterial stiffness in obese population (53). In brief, metabolic status plays an important role in arterial stiffness.

On the other hand, arterial stiffness is also an important factor of cardiometabolic health. A community-based cohort study (23) demonstrated that arterial stiffness precedes diabetes, and arterial stiffness changes may precede changes in fasting plasma glucose. Muhammad et al. (22) found that increased arterial stiffness, as determined by cfPWV, is significantly associated with an increased risk of developing diabetes, possibly through microvascular dysfunction and impaired tissue perfusion. Furthermore, a long-term, large, multicenter prospective cohort study (54) found that increased arterial stiffness measured by baPWV could predict the risk of all-cause and cause-specific death in type 2 diabetes. Genetic evidence (55) also suggests that arterial stiffness is associated with an increased risk of diabetes in a dose-response manner, which is partially reinforced by a high genetic susceptibility to diabetes. However, there is limited evidence on the reciprocal associations between arterial stiffness and lipid profiles.

A birth cohort study (27) suggested that arterial stiffness may be a risk factor for hyperinsulinemia and insulin resistance in young adulthood. Higher baseline cfPWV is associated with higher fasting insulin, insulin resistance, and beta cell function, and cfPWV increase is directly related with the 7-year progression in HDL-C and triglycerides in youth. Arterial stiffness is also a significant mediator for high total cholesterol level (40). Our study highlighted that high baPWV is positively associated with the incidence of dyslipidemia in the general population. In this current study, the cumulative incidence was relatively high (34.2%), which could possibly be explained by the high proportion of excessive BMI and smoking, and low rate of regular physical activity of the population (**Table 1**). We also found that baPWV changes were significantly associated with lipids parameters progression, although the correlation coefficients were relatively mild, which indicated that dyslipidemia is a multi-factorial affected metabolic status including arterial stiffness. People of new-onset and persistent high baPWV were significantly associated with an increased risk of dyslipidemia, but not the remitted baPWV. The prevalence of hypertension and use of antihypertensive medication were higher among the remitted group (**Supplementary Table S4**), which could partially explain the reduced arterial stiffness level given the protective effect of antihypertensive medication on vascular stiffness (56, 57).

Several potential mechanisms that might explain our findings. Under normal conditions, most of the lipids can be leaked into the arterial wall by the arterial intima and then drained out by the lymphatic vessels in the arterial epithelium. When the body’s regulation of lipid metabolism malfunctions, the concentration of cholesterol and triglycerides in the lipid composition increases in hyperlipidemia, causing damage to the inner wall of the artery (58). Studies have shown that arterial stiffness plays a central role in a vicious cycle of hemodynamic dysfunction characterized by excessive pulsation, ultimately leading to heart failure, impaired coronary perfusion, chronic kidney disease, cerebrovascular disease and other chronic diseases (59). In addition, vascular endothelial dysfunction refers to the abnormal function of endothelial cells after stimulation by risk factors such as hyperlipidemia and is generally considered to be the initiating link of atherosclerosis. Endothelial injury, which can weaken its physiological function, increases the risk of lipid infiltration and leads to a series of pathological changes such as decreased biological activity of nitric oxide, smooth cell migration, plaque instability, and thrombosis (60), and studies have shown that endothelial dysfunction is also associated with arterial stiffness. However, the mechanism between arterial stiffness and dyslipidemia needs further exploration.

Mendelian randomization studies (61) have shown that there is a bidirectional causal relationship between blood pressure and arterial stiffness, and the genetic basis of blood pressure is mediated not only by genes that regulate blood pressure, but also by genes that affect arterial stiffness. Saeed et al (62) found that there appears to be a bidirectional association between arterial stiffness and COVID-19 severity. The pre-existing atherosclerosis is an independent risk factor for COVID-19 severity, while endothelial cell damage caused by direct coronavirus invasion or inflammatory cytokine storms is also considered to be a key factor in the pathogenesis of COVID-19-associated arterial stiffness. There are few studies on the bidirectional causality between arterial stiffness and metabolic health, and our study indicated a bidirectional causality between arterial stiffness and dyslipidemia, which needs further research.

The effect of dyslipidemia on arterial stiffness has been validated on previous studies. In this current study, we supplemented the evidence about the association of arterial stiffness with the incidence of dyslipidemia using a population-based cohort design. Several limitations of the current study should be acknowledged. First, there are many indicators to comprehensively evaluate the arterial stiffness level, including baPWV and carotid femoral PWV (cfPWV). The American Heart Association research report (63) pointed out that cfPWV is a credible standard for checking the degree of arterial stiffness. The cfPWV data was not available in this study and we were unable to compare the results between baPWV and cfPWV. However, many studies have showed that there was a high consistency between baPWV and cfPWV, and the measurement of baPWV could enhance the risk prediction of cardiovascular disease. Second, this study demonstrated an observational association between arterial stiffness and dyslipidemia, but not causal association due to the study design. Further studies are needed to explore the causal and temporal relationships between arterial stiffness and dyslipidemia.

## 5 Conclusions

In short, our study indicated that elevated baPWV level is significantly associated with development of dyslipidemia among the general population, which needs further investigation and validation in other populations.

## Data availability statement

The raw data supporting the conclusions of this article will be made available by the authors, without undue reservation.

## Ethics statement

The studies involving humans were approved by Beijing Xiaotangshan Hospital Ethics Committee. The studies were conducted in accordance with the local legislation and institutional requirements. The participants provided their written informed consent to participate in this study.

## Author contributions

Literature search: HW, ZM. Study conception and design: HS, YQ. Data collection: PX, JZ. Data analysis and interpretation: HW, TZ. Manuscript writing and reviewing: HW, CC. Study supervision: XQ, CC. All authors contributed to the article and approved the submitted version.
