# Supplementary material for: Association of baseline and dynamic arterial stiffness status with dyslipidemia: a cohort study
Source: Front Endocrinol (Lausanne). 2023 Nov 23;14:1243673. doi: 10.3389/fendo.2023.1243673 (PMC10704037; doi:10.3389/fendo.2023.1243673)
Supplement: Supplementary file 1 [file Table_1.doc]

Table S1: Characteristics of 4362 participants according to baPWV level.

|  | BaPWV <1400 cm/s | BaPWV ≥1400 cm/s | P value |
| --- | --- | --- | --- |
| Participants, No. | 2182 | 2180 |  |
| Age, mean (SD), y | 47.79(9.35) | 63.28(13.47) |  |
| -39 y, n (%) | 374(17.1) | 63(2.9) | <0.001 |
| 40-59 y, n (%) | 1604(73.5) | 876(40.2) |  |
| 60- y, n (%) | 204(9.3) | 1241(56.9) |  |
| Sex, n (%) |  |  |  |
| Male, n (%)  Female, n (%) | 1325(60.7)  857(39.3) | 1668(76.5)  512(23.5) | <0.001 |
| BMI, mean (SD), Kg/m^2 a^ | 24.61(3.02) | 25.44(3.10) | <0.001 |
| Obesity, n (%) | 275(12.8) | 406(19.0) | <0.001 |
| Physical activity (n, %) ^b^ | 1041(47.7) | 1041(47.8) | 1 |
| Smoking status (n, %) |  |  |  |
| Never | 1648(75.5) | 1650(75.7) | 0.946 |
| Former | 261(12.0) | 254(11.7) |  |
| Current | 273(12.5) | 276(12.7) |  |
| Drinking status (n, %) |  |  |  |
| Not current drinking | 753(34.5) | 792(36.3) | 0.22 |
| Self-reported hypertension (n, %) | 74(3.4) | 278(12.8) | <0.001 |
| Self-reported diabetes (n, %) | 22(1.0) | 97(4.4) | <0.001 |
| MAP, mean (SD), mmHg ^c^ | 100.09(10.96) | 112.29(12.84) | <0.001 |
| Fasting glucose, mean (SD), mmol/L | 5.19(0.79) | 5.75(1.25) | <0.001 |
| Triglycerides, median [IQR], mg/dl | 96.57[72.65,131.13] | 105.43[78.85,137.33] | <0.001 |
| HDL-C, median [IQR], mg/dl | 50.64[44.46,58.76] | 48.33[42.91,56.44] | <0.001 |
| LDL-C, median [IQR], mg/dl | 117.91[98.97,134.15] | 115.98[96.26,131.83] | 0.015 |
| Total cholesterol, median [IQR], mg/dl | 182.09[163.15,199.49] | 179.38[160.44,198.71] | 0.008 |
| NonHDL-C, median [IQR], mg/dl ^d^ | 129.51[109.79,147.29] | 129.12[109.41,146.91] | 0.585 |
| Remnant cholesterol, median [IQR], mg/dl ^e^ | 19.46[16.11,24.23] | 20.49[16.95,24.78] | <0.001 |
| baPWV, median [IQR], cm/s | 1266[1188,1331] | 1621[1486,1851] | <0.001 |
| Incident dyslipidemia (n, %) | 640(29.3) | 850(39.0) | <0.001 |

Abbreviations: SD, standard deviation; IQR, interquartile range; BMI, body mass index; MAP, mean arterial pressure; baPWV, brachial-ankle pulse wave velocity; LDL-C, low-density lipoprotein cholesterol; HDL-C, high-density lipoprotein cholesterol.

Table S2: Association between elevated baPWV and the development of dyslipidemia stratified by age.

|  | <60 years | | ≥60 years | |
| --- | --- | --- | --- | --- |
|  | adjusted HR (95% CI) | *P* value | adjusted HR (95% CI) | *P* value |
| baPWV <1400 cm/s | Ref |  |  |  |
| baPWV ≥1400 cm/s | 1.194 (1.036-1.376) | .01 | 1.310 (1.024-1.675) | .03 |

Abbreviations: CI, confidence interval; baPWV, brachial-ankle pulse wave velocity.

Analyses were adjusted for age, sex, obesity, fasting glucose, diabetes, hypertension, smoking, drinking and physical activity.

Table S3: Association between baseline baPWV and the development of dyslipidemia defined by the use of lipid-lowering therapy.

|  | Model 1 | | | Model 2 | | |
| --- | --- | --- | --- | --- | --- | --- |
|  | HR | 95% CI | P value | HR | 95% CI | P value |
| baPWV <1400 cm/s | Ref |  |  |  |  |  |
| baPWV ≥1400 cm/s | 1.648 | 1.250-2.172 | <0.001 | 1.636 | 1.233-2.171 | 0.001 |
| Quartile groups |  |  |  |  |  |  |
| -1266 cm/s | Ref |  |  |  |  |  |
| 1267-1399 cm/s | 1.443 | 0.951-2.190 | 0.08 | 1.452 | 0.946-2.229 | 0.09 |
| 1400-1620 cm/s | 2.208 | 1.500-3.249 | <0.001 | 2.191 | 1.472-3.262 | <0.001 |
| 1621- cm/s | 1.756 | 1.151-2.678 | 0.009 | 1.777 | 1.149-2.749 | 0.01 |

Abbreviations: CI, confidence interval; baPWV, brachial-ankle pulse wave velocity.

model 1: adjusted for age and sex; model 2: adjusted for age, sex, obesity, fasting glucose, diabetes, hypertension, smoking, drinking and physical activity.

Table S4: Characteristics according to baseline and follow-up baPWV status.

|  | Persistent low baPWV | New-onset high baPWV | Remitted low baPWV | Persistent high baPWV |
| --- | --- | --- | --- | --- |
| Participants, No. | 1602 | 580 | 216 | 1964 |
| Age, mean (SD), years | 45.83(8.70) | 53.22(8.91) | 49.93(7.62) | 64.75(13.17) |
| Male, n (%) | 927(57.9) | 398(68.6) | 160(74.1) | 1508(76.8) |
| BMI, mean (SD), kg/m^2^ | 24.45(3.01) | 25.06(2.99) | 25.48(3.35) | 25.43(3.07) |
| Obesity, n (%) | 176(11.2) | 99(17.3) | 41(19.3) | 365(18.9) |
| Physical activity (n, %) | 743(46.4) | 298(51.4) | 109(50.5) | 932(47.5) |
| Smoking status (n, %) |  |  |  |  |
| Never | 1225(76.5) | 423(72.9) | 162(75.0) | 1488(75.8) |
| Former | 190(11.9) | 71(12.2) | 28(13.0) | 226(11.5) |
| Current | 187(11.7) | 86(14.8) | 26(12.0) | 250(12.7) |
| Current drinking (n, %) | 1035(64.6) | 394(67.9) | 158(73.1) | 1230(62.6) |
| Self-reported hypertension, n (%) | 38(2.4) | 36(6.2) | 17(7.9) | 261(13.3) |
| Anthypertensive medication, n (%) | 32(2.0) | 32(5.5) | 15(6.9) | 232(11.8) |
| Self-reported diabetes, n (%) | 11(0.7) | 11(1.9) | 3(1.4) | 94(4.8) |
| Fasting glucose, mean (SD), mmol/L | 5.15(0.75) | 5.33(0.87) | 5.46(1.12) | 5.78(1.26) |
| Triglyceride, median [IQR], mg/dl | 95.69[71.77,128.47] | 106.32[74.64,139.10] | 114.29[81.96,141.32] | 104.55[78.85,136.66] |
| Total cholesterol, median [IQR], mg/dl | 182.09[163.15,199.10] | 182.09[164.40,200.26] | 183.64[168.17,200.84] | 179.00[159.67,198.33] |
| LDL-C, median [IQR], mg/dl | 117.91[98.97,133.76] | 117.91[99.07,135.60] | 120.62[106.32,133.18] | 115.21[95.49,131.83] |
| HDL-C, median [IQR], mg/dl | 51.42[44.85,59.15] | 49.48[43.30,57.51] | 48.33[43.10,57.02] | 48.33[42.91,56.44] |
| nonHDL-C, median [IQR], mg/dl | 129.12[109.02,146.32] | 130.48[111.44,148.74] | 132.99[115.40,148.45] | 128.74[108.25,146.91] |
| Remnant-C, median [IQR], mg/dl | 19.28[15.99,23.54] | 20.48[16.49,25.10] | 21.33[17.43,25.16] | 20.36[16.94,24.74] |
| baPWV, median [IQR], cm/s | 1238[1170,1305] | 1319[1269,1363] | 1443[1421,1482] | 1656[1512,1881] |
| Incident dyslipidemia (n, %) | 414(25.8) | 226(39.0) | 76(35.2) | 774(39.4) |

Abbreviations: baPWV, brachial-ankle pulse wave velocity; LDL-C, low-density lipoprotein cholesterol; HDL-C, high-density lipoprotein cholesterol.

Table S5: Regression analysis between baPWV changes and the progression of lipid parameters.

|  | β (95% CI) | | | |
| --- | --- | --- | --- | --- |
|  | model 1 | P value | model 2 | P value |
| Triglyceride change, mg/dl | 1.189(0.211-2.167) | 0.017 | 1.03(0.025-2.036) | 0.045 |
| Total cholesterol change, mg/dl | 0.899(0.441-1.356) | <0.001 | 0.827(0.369-1.284) | <0.001 |
| LDL-C change, mg/dl | 0.917(0.478-1.357) | <0.001 | 0.862(0.42-1.304) | <0.001 |
| HDL-C change, mg/dl | 0.213(0.05-0.376) | 0.01 | 0.233(-0.069-0.306) | 0.055 |
| NonHDL-C change, mg/dl | 0.685(0.219-1.152) | 0.004 | 0.594(0.13-1.058) | 0.012 |
| Remnant-C, mg/dl | 0.174(0.058-0.291) | 0.003 | 0.153(0.033-0.272) | 0.012 |

Abbreviations: CI, confidence interval; baPWV, brachial-ankle pulse wave velocity; LDL-C, low-density lipoprotein cholesterol; HDL-C, high-density lipoprotein cholesterol; Remnant-C, remnant cholesterol.

model 1: adjusted for age and sex; model 2: adjusted for age, sex, obesity, fasting glucose, self-reported diabetes, self-reported hypertension, smoking, drinking, and physical activity.
